# Associations between work characteristics and osteoarthritis: A cross-sectional study of 285,947 UK Biobank participants

**DOI:** 10.1016/j.ocarto.2025.100565

**Published:** 2025-01-10

**Authors:** A. Hashmi, S. Scott, M. Jung, Q.-J. Meng, J.H. Tobias, R.A. Beynon, B.G. Faber

**Affiliations:** a Musculoskeletal Research Unit, University of Bristol, UK; b Medical Research Council Integrative Epidemiology Unit, University of Bristol, UK; c Wellcome Centre for Cell-Matrix Research, Faculty of Biology, Medicine and Health, University of Manchester, UK

**Keywords:** Epidemiology, Knee osteoarthritis, Hip osteoarthritis, Occupational risk, Shift work, Physical work

## Abstract

**Objectives:**

Shift work-induced circadian rhythm disruption has been identified as a risk factor for specific diseases. Additionally, physically demanding work has been linked to osteoarthritis. This study investigated the independent associations of shift work and physical work with risk of osteoarthritis.

**Design:**

UK Biobank participants completed questionnaires detailing their employment status, including shift work, night shifts, heavy manual work and prolonged non-sedentary work. Responses were categorised into binary and categorical variables. Knee and hip osteoarthritis diagnoses were extracted from hospital records and osteoarthritis (any site) was self-reported. Logistic regression models, adjusted for age, sex, BMI, Townsend Deprivation Index and other work factors, were used to investigate the relationships between work characteristics and osteoarthritis outcomes.

**Results:**

This study included 285,947 participants (mean age 52.7 years; males 48.0 ​%). Shift work and night shifts were associated with knee osteoarthritis (fully adjusted OR: 1.12 [95 ​% CI:1.07–1.17] and 1.12 [1.04–1.20], respectively), and self-reported osteoarthritis but there was little evidence of an association with hip osteoarthritis (1.01 [0.95–1.08] and 1.03 [0.93–1.14]). Heavy manual work and prolonged non-sedentary work were associated with increased risk of all osteoarthritis outcomes.

**Conclusions:**

Shift work showed independent associations with knee osteoarthritis and self-reported osteoarthritis but not hip osteoarthritis, suggesting circadian rhythm dysfunction may play a role in knee osteoarthritis pathogenesis. Heavy manual work and prolonged non-sedentary work were associated with all outcomes, with stronger associations in knee osteoarthritis, possibly reflecting the knee’s higher susceptibility to biomechanical stress. Further research is needed to explore workplace interventions for reducing these risks.

## 1 Introduction

Osteoarthritis is the 7th leading cause of years lived with disability worldwide, particularly in those aged over 70, with the knee and hip joints being the most affected [1,2]. The global disease burden is increasing due to an upsurge in prevalence, driven by an ageing demographic and a growing incidence of obesity [3]. Risk factor identification has the potential to alleviate this burden by helping prevent disease onset and progression. Given that individuals in the UK spend an average of 1532 hours at work annually [4], the workplace environment emerges as an important area for uncovering such risk factors. Notably, shift work-induced circadian disruption has received growing interest, with over 20 ​% of European workers engaged in shift work in 2015 [5].

Circadian (24-hourly) rhythm has been shown to play a role in maintaining joint tissue homeostasis [6]. Circadian rhythm is generated by a network of clock genes and proteins that rhythmically regulate their own expression and that of other tissue-specific target genes, through a transcription-translation feedback loop [7]. Notably, genes involved in extracellular matrix turnover and chondrocyte differentiation demonstrate circadian rhythmicity, with clock genes implicated in this regulation [8]. In human osteoarthritis models, disruptions in clock gene function have been linked to increased expression of cartilage catabolic genes [9,10], while environmental disturbances of circadian rhythms have been shown to induce osteoarthritis-like alterations in the mouse knee joint [11].

Shift work is a known disruptor of circadian rhythms, by causing a misalignment in the sleep-wake cycle, raising the possibility it might influence osteoarthritis risk [12]. Retrospective cohort studies of retired Chinese workers have revealed positive associations between shift work and risk of osteoarthritis, compared with daytime workers [13,14]. However, the generalisability of these findings to other populations may be limited. These associations have not yet been fully explored in the UK, where men and women engaging in night shift work are more likely to perform physically demanding tasks [15], a known risk factor for osteoarthritis [16]. For example, a study of 327 participants found a 3-fold increase in odds of radiographic knee osteoarthritis in workers with the highest cumulative occupational physical load [17], with similar findings seen in other small cohort studies [18,19]. While research is largely focused on the knee [20], some comparable associations have been seen for the hip [21,22]. Therefore, when exploring the association between shift work and osteoarthritis, it is important to consider the influence of work physicality as a possible confounding factor. Further work is also justified to provide more updated understanding of the associations between physically demanding work and osteoarthritis, in a larger cohort.

This study aimed to investigate the relationships between work characteristics and the prevalence of osteoarthritis within the UK Biobank (UKB) study. Specifically, our objectives were to explore the independent associations between shift work and the prevalence of hospital-diagnosed knee and hip osteoarthritis, as well as self-reported osteoarthritis, whilst considering the influence of work physicality. Additionally, we sought to assess the independent influence of work physicality on risk of osteoarthritis and to identify any sex differences in these relationships. By addressing these questions, our study aimed to offer insights into the associations between these work characteristics and joint health, with the goal of informing the development of future interventions and employment pattern policies, to alleviate the burden of osteoarthritis.

## 2 Materials and methods

### 2.1 Population

UKB is a longitudinal, population-based cohort study, that commenced in 2006, aiming to recruit over 500,000 individuals aged 40–69 from across the UK. Participants underwent extensive physical and lifestyle assessment, including questionnaires on socio-demographic and environmental factors. Consent was given for linkage to electronic healthcare records, which enabled participant follow-up [23]. UKB obtained ethical approval from the National Information Governance Board for Health and Social Care and Northwest Multi-centre Research Ethics Committee (11/NW/0382). All participants provided informed consent for the collection and use of their data.

### 2.2 Exposures

All UKB participants were asked about their work patterns via a touchscreen questionnaire at baseline, with a subsample of participants (∼20 ​%) having the opportunity to complete this again at one of three follow up appointments. Considering most respondents in this study only answered these questions at baseline (89 ​%), this information was collapsed into one set of variables. The questions included “*Does your work involve shift work?*”, defined as a work schedule outside the routine daytime working hours of 09:00 to 17:00. Participants responded according to a four-point Likert scale: “Never/Rarely”, “Sometimes”, “Usually” or “Always”. Those who answered sometimes, usually or always were subsequently asked “*Does your work involve night shifts?*”, defined as a work schedule that involves working during the regular sleeping hours, for example from 00:00 to 06:00. Participants answered according to the same four responses. For work physicality, participants were asked, “*Does your work involve heavy manual or physical work?*” and “*Does your work involve walking or standing for most of the time?*”. Heavy manual work was defined as employment involving the use of heavy objects and tools. Prolonged non-sedentary work was defined as work that requires prolonged periods of walking or standing. Again, responses followed the same four-category scale.

Categorical variables for the work exposures were created and coded; (1) never/rarely, (2) sometimes, (3) usually or (4) always. From this, a binary exposure variable was generated: (0) never/rarely and sometimes or (1) usually and always. If a participant answered more than once, their highest response was selected. Those who did not know or preferred not to answer were excluded from analyses.

### 2.3 Self-reported outcomes

Self-reported diagnoses of generalised, non-site-specific osteoarthritis were collected through verbal interviews at baseline, with additional data available for a subsample of participants across multiple timepoints, similar to the employment questionnaire. A binary variable was created to classify participants: those who reported a diagnosis of osteoarthritis at any timepoint were coded as “1”, while those who did not report a diagnosis of osteoarthritis at any point were coded as “0”.

### 2.4 Diagnosed outcomes

Hospital diagnosed knee osteoarthritis and hip osteoarthritis cases were identified through linkage to hospital episode statistics, which use the International Classification of Diseases 9th and 10th revision codes. Specifically, cases were identified using codes adopted from Zengini et al. [24] (Supplementary Table 1). The linkage of all UKB participants was conducted both prospectively and retrospectively from baseline. Records were available from April 1, 1997, and the data was downloaded from the UKB Showcase in August 2023, encompassing information until the end of October 2022.

### 2.5 Statistical analysis

Descriptive statistics summarised the baseline population characteristics, presented as means, standard deviations and ranges for continuous variables and as frequencies for categorical and binary variables. Logistic regression models examined the associations between each work exposure (shift work, night shifts, heavy manual work and prolonged non-sedentary work) and each osteoarthritis outcome (knee, hip and self-reported). Results are presented as odds ratios with 95 ​% confidence intervals. Models were adjusted for confounders (age, sex, and Townsend Deprivation Index (TDI)) and potential mediators (Body Mass Index). Only individuals with complete covariate data were included in this study (Supplementary Fig. 1). The TDI is a postcode-based measure of socioeconomic deprivation, where higher measures indicate increased deprivation [25]. TDI was chosen as a proxy for other deprivation-related factors. However, given the strong influence of education on employment type, a further model was run to include this as a sensitivity analysis.

Models of shift work exposures were adjusted accordingly: (1) unadjusted, (2) age and sex, (3) age, sex, BMI and TDI, (4) age, sex, BMI, TDI and both work physicality variables. Models using work physicality exposures were similarly adjusted except for model 4, which was adjusted for age, sex, BMI, TDI, shift work and the other work physicality variable. Age, BMI and TDI were incorporated into the models as continuous variables. Shift work and night shifts were not mutually adjusted due to direct classification overlap. The effect of exposure frequency on risk of osteoarthritis was explored using categorial work exposure variables, which used the “never” group as the comparator. Combined sex analyses were supplemented by sex-stratified analyses. Sex interaction terms were also examined for the associations between the work-based exposures and osteoarthritis outcomes. All statistical analyses were conducted using STATA version 18 (StataCorp, College Station, TX, USA). A pre-specified analysis plan is included as a Supplementary File.

## 3 Results

### 3.1 Baseline characteristics

In total, 285,947 participants (mean age 52.7, SD: 7.1 years, range: 38–71 years), were included in the analysis (Table 1). There were 137,154 (48.0 ​%) males and 148,793 (52.0 ​%) females. Knee osteoarthritis and hip osteoarthritis were diagnosed in 18,578 (6.5 ​%) and 10,698 (3.7 ​%) individuals, respectively. Non-site-specific osteoarthritis was self-reported in 16,407 (5.7 ​%) individuals and only 5601 (34 ​%) of these participants had a concurrent diagnosis of knee and/or hip osteoarthritis. Knee osteoarthritis was more common among males with a prevalence of 9623 (7.0 ​%) compared to females (8955 [6.0 ​%]). Hip osteoarthritis and self-reported osteoarthritis were more common among females (5895 [4.0 ​%] and 10,018 [6.7 ​%], respectively) than males (4803 [3.5 ​%] and 6389 [4.7 ​%], respectively). Shift work, nights shifts and heavy manual work were more commonly reported among males, whereas prolonged non-sedentary work had a similar prevalence among males and females (Table 1).

**Table 1:** Descriptive statistics of study population.

	Combined	Male	Female
	Mean [SD, Range]	Mean [SD, Range]	Mean [SD, Range]
Age at baseline	52.7 [7.1, 38–71]	53.2 [7.4, 38–70]	52.3 [6.8, 40–71]
BMI	27.3 [4.7, 13.6–74.7]	27.8 [4.1, 14.9–63.3]	26.8 [5.1, 13.6–74.7]
TDI	−1.3 [3.0, −6.3 to 11.0]	−1.4 [3.0, −6.3 to 10.5]	−1.3 [3.0, −6.3 to 11.0]
**Categorical exposures**	**N [%]**	**N [%]**	**N [%]**
**Shift work**			
Never/Rarely	235,136 [82.2]	109,362 [79.7]	125,774 [84.5]
Sometimes	21,855 [7.6]	12,043 [8.8]	9812 [6.6]
Usually	6271 [2.2]	3217 [2.4]	3054 [2.1]
Always	22,685 [7.9]	12,532 [9.1]	10,153 [6.8]
**Night shifts**			
Never/Rarely	260,177 [91.0]	121,194 [88.4]	138,983 [93.4]
Sometimes	14,432 [5.1]	8929 [6.5]	5503 [3.7]
Usually	3968 [1.4]	2512 [1.8]	1456 [1.0]
Always	7370 [2.6]	4519 [3.3]	2851 [1.9]
**Heavy manual work**			
Never/Rarely	183,796 [64.3]	81,022 [59.1]	102,774 [69.1]
Sometimes	62,355 [21.8]	31,693 [23.1]	30,662 [20.6]
Usually	20,010 [7.0]	12,257 [8.9]	7753 [5.2]
Always	19,786 [6.9]	12,182 [8.9]	7604 [5.1]
**Non-sedentary work**			
Never/Rarely	97,507 [34.1]	44,043 [32.1]	53,464 [35.9]
Sometimes	87,981 [30.8]	43,956 [32.1]	44,025 [29.6]
Usually	43,023 [15.1]	21,470 [15.7]	21,553 [14.5]
Always	57,436 [20.1]	27,685 [20.2]	29,751 [20.0]
**Outcomes**			
Knee osteoarthritis	18,578 [6.5]	9623 [7.0]	8955 [6.0]
Hip osteoarthritis	10,698 [3.7]	4803 [3.5]	5895 [4.0]
Self-reported osteoarthritis	16,407 [5.7]	6389 [4.7]	10,018 [6.7]
**Total number of observations**	**285,947 [100.0]**	**137,154 [48.0]**	**148,793 [52.0]**

Abbreviations: BMI – body mass index, TDI – Townsend Deprivation Index.

### 3.2 Knee osteoarthritis

Combined sex analyses (models 1–3) demonstrated consistent associations between binary shift work and knee osteoarthritis (model 3: odds ratio (OR) 1.29 [95 ​% CI: 1.23–1.35]), with a similar trend for night shifts. Sex interaction terms did not provide significant evidence of an interaction (*p* ​< ​0.1), except for the unadjusted association with shift work (Table 2). In sex stratified analyses, the effect estimates were broadly comparable between males and females (Table 2, Supplementary Table 2). Work physicality exposures exhibited stronger associations, with the point estimates for heavy manual work (model 3: 1.58 [1.52–1.64]) slightly higher than prolonged non-sedentary work (model 3: 1.48 [1.43–1.52]). For both exposures, sex interactions were seen in all models with sex stratification showing they were more strongly associated with knee osteoarthritis in males compared to females (Table 2, Supplementary Fig. 2).

**Table 2:** Logistic regression results showing the associations between binary work exposures and knee osteoarthritis in combined and sex-stratified analyses.

Knee osteoarthritis	Model 1		Model 2		Model 3		Model 4	
	OR [95 ​% CI]	P	OR [95 ​% CI]	P	OR [95 ​% CI]	P	OR [95 ​% CI]	*P*
**Combined**								
Shift work	1.31 [1.25–1.37]	8.87 ​× ​10^−32^^a^	1.42 [1.36–1.49]	6.30 ​× ​10^−52^	1.29 [1.23–1.35]	3.73 ​× ​10^−27^	1.12 [1.07–1.17]	3.87 ​× ​10^−6^^a^
Night shifts	1.32 [1.23–1.42]	1.26 ​× ​10^−15^	1.46 [1.36–1.57]	6.61 ​× ​10^−27^	1.30 [1.21–1.39]	3.39 ​× ​10^−13^	1.12 [1.04–1.20]	1.75 ​× ​10^−3^^a^
Heavy manual work	1.55 [1.49–1.61]	2.00 ​× ​10^−113^^a^	1.59 [1.53–1.65]	3.00 ​× ​10^−122^^a^	1.58 [1.52–1.64]	4.00 ​× ​10^−114^^a^	1.30 [1.24–1.36]	5.76 ​× ​10^−30^^a^
Non-sedentary work	1.48 [1.44–1.53]	2.00 ​× ​10^−146^^a^	1.46 [1.41–1.50]	7.00 ​× ​10^−131^^a^	1.48 [1.43–1.52]	3.00 ​× ​10^−135^^a^	1.32 [1.28–1.37]	5.30 ​× ​10^−54^^a^
**Males**								
Shift work	1.24 [1.16–1.31]	9.11 ​× ​10^−12^	1.39 [1.31–1.48]	6.79 ​× ​10^−26^	1.32 [1.24–1.40]	1.09 ​× ​10^−17^	1.14 [1.07–1.21]	8.34 ​× ​10^−5^
Night shifts	1.24 [1.14–1.35]	1.14 ​× ​10^−6^	1.43 [1.31–1.56]	1.41 ​× ​10^−15^	1.34 [1.23–1.46]	1.14 ​× ​10^−10^	1.15 [1.05–1.26]	2.14 ​× ​10^−3^
Heavy manual work	1.65 [1.57–1.73]	4.70 ​× ​10^−93^	1.73 [1.65–1.81]	2.00 ​× ​10^−108^	1.76 [1.68–1.85]	3.00 ​× ​10^−112^	1.38 [1.30–1.46]	6.18 ​× ​10^−28^
Non-sedentary work	1.66 [1.59–1.73]	6.00 ​× ​10^−126^	1.64 [1.58–1.71]	3.00 ​× ​10^−119^	1.69 [1.62–1.76]	3.00 ​× ​10^−127^	1.45 [1.38–1.52]	1.10 ​× ​10^−46^
**Females**								
Shift work	1.38 [1.29–1.47]	1.65 ​× ​10^−20^	1.45 [1.36–1.55]	7.40 ​× ​10^−27^	1.26 [1.18–1.36]	4.59 ​× ​10^−11^	1.14 [1.06–1.22]	6.78 ​× ​10^−4^
Night shifts	1.39 [1.24–1.55]	9.06 ​× ​10^−9^	1.49 [1.33–1.67]	3.30 ​× ​10^−12^	1.23 [1.10–1.38]	4.09 ​× ​10^−4^	1.09 [0.97–1.23]	0.14
Heavy manual work	1.32 [1.24–1.41]	1.48 ​× ​10^−17^	1.38 [1.29–1.47]	3.82 ​× ​10^−22^	1.31 [1.23–1.40]	6.39 ​× ​10^−16^	1.12 [1.04–1.21]	1.97 ​× ​10^−3^
Non-sedentary work	1.31 [1.25–1.37]	1.26 ​× ​10^−33^	1.28 [1.22–1.33]	1.32 ​× ​10^−27^	1.28 [1.23–1.34]	1.62 ​× ​10^−27^	1.22 [1.16–1.28]	1.76 ​× ​10^−14^

Adjusted accordingly: Model 1 – unadjusted, Model 2 – age and sex, Model 3 – age, sex, BMI and TDI, Model 4 – age, sex, BMI, TDI and other employment factors.

a Denotes a sex interaction term with *P*-value <0.1.

Mutual adjustment (model 4) attenuated the associations between binary work characteristics and knee osteoarthritis. In combined sex analyses, shift work and night shifts continued to be associated with knee osteoarthritis (shift work: OR 1.12 [95 ​% CI: 1.07–1.17], night shifts: 1.12 [1.04–1.20]). Unlike models 1–3, sex interactions were noted in the fully adjusted models for both shift work and night shifts (Table 2). Upon sex stratification, night shifts remained associated with knee osteoarthritis in males (1.15 [1.05–1.26]) but not females (1.09 [0.97–1.23]). In combined sex analyses, work physicality remained strongly associated with knee osteoarthritis (heavy manual work: 1.30 [1.24–1.36], prolonged non-sedentary work: 1.32 [1.28–1.37]). Sex interactions persisted, with stronger associations between work physicality and knee osteoarthritis in males than in females (Table 2, Supplementary Fig. 2). Similar effect estimates were seen in sensitivity analyses that included educational attainment as a covariate (Supplementary Table 2).

**Fig. 1:**
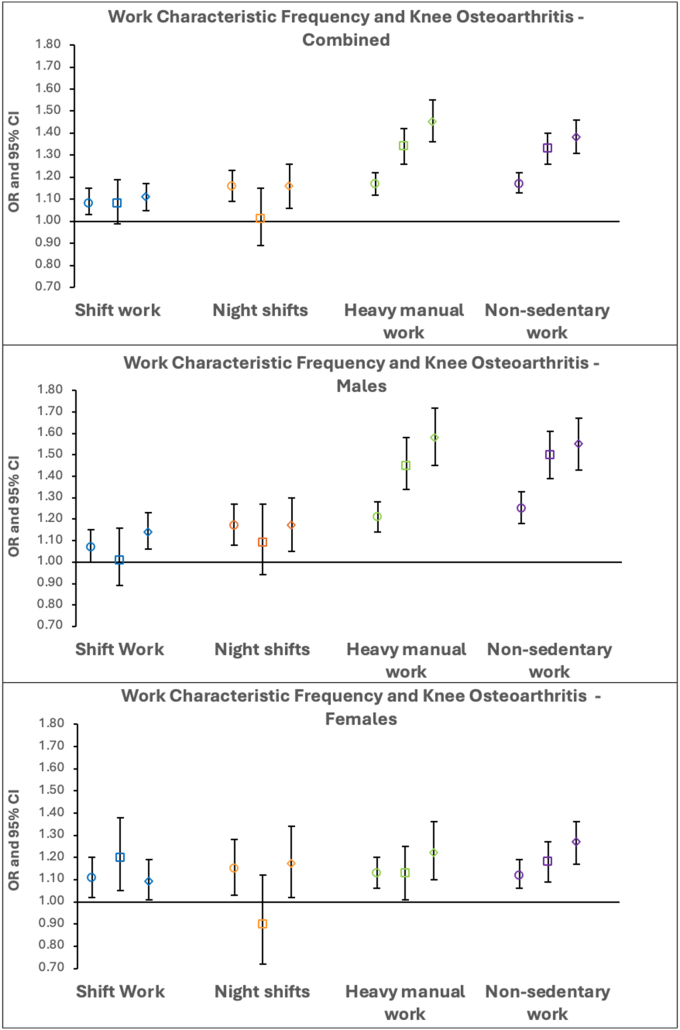
**Logistic regression results for the associations between categorical work characteristic frequency and knee osteoarthritis in fully-adjusted combined and sex-stratified analyses.** Odds ratios with the comparator group “never” are displayed with 95 ​% CIs. Different shapes represent the various employment intensities - circle: “sometimes”, square: “usually” and diamond: “always”.

When work type was assessed as a categorical variable (1–4), fully adjusted models showed no evidence of a progressive positive association between more frequent shift work or nights shifts and knee osteoarthritis. Similar results were seen in sex stratified analyses (Fig. 1, Supplementary Table 3). In contrast, a distinct progressive association emerged between frequency of heavy manual work and prolonged non-sedentary work with knee osteoarthritis. The progressive trend was reflected in results for males and females, but effect sizes were larger in males (Fig. 1, Supplementary Table 3).

### 3.3 Hip osteoarthritis

Combined sex analyses (models 1–3) demonstrated little evidence of associations between binary shift work and hip osteoarthritis (model 3: OR 1.08 [95 ​% CI: 1.01–1.15]. A similar lack of association was seen in analyses of night shifts. No sex interactions were seen for either exposure and sex-stratification indicated little differences between males and females (Table 3, Supplementary Fig. 3). More robust associations were seen between work physicality and hip osteoarthritis, although weaker than those for knee osteoarthritis. Sex interactions were only evident for prolonged non-sedentary work. Sex-stratified analyses saw stronger associations between prolonged non-sedentary work and hip osteoarthritis in males than females (Table 3, Supplementary Fig. 3).

**Table 3:** Logistic regression results showing the associations between binary work exposures and hip osteoarthritis in combined and sex-stratified analyses.

Hip osteoarthritis	Model 1		Model 2		Model 3		Model 4	
	OR [95 ​% CI]	P	OR [95 ​% CI]	P	OR [95 ​% CI]	P	OR [95 ​% CI]	P
**Combined**								
Shift work	0.99 [0.93–1.05]	0.74	1.12 [1.05–1.20]	5.60 ​× ​10^−4^	1.08 [1.01–1.15]	0.03	1.01 [0.95–1.08]	0.74
Night shifts	0.97 [0.88–1.07]	0.54	1.16 [1.04–1.28]	5.24 ​× ​10^−3^	1.10 [0.99–1.21]	0.08	1.03 [0.93–1.14]	0.59^a^
Heavy manual work	1.13 [1.07–1.19]	1.00 ​× ​10^−5^	1.22 [1.15–1.29]	1.55 ​× ​10^−12^	1.22 [1.15–1.28]	2.87 ​× ​10^−12^	1.13 [1.06–1.20]	1.32 ​× ​10^−4^
Non-sedentary work	1.19 [1.14–1.24]	5.10 ​× ​10^−18^^a^	1.16 [1.11–1.21]	3.53 ​× ​10^−13^^a^	1.17 [1.12–1.21]	5.17 ​× ​10^−14^^a^	1.12 [1.07–1.17]	1.39 ​× ​10^−6^^a^
**Males**								
Shift work	0.96 [0.88–1.05]	0.39	1.12 [1.02–1.23]	0.01	1.09 [0.99–1.19]	0.08	1.02 [0.93–1.12]	0.67
Night shifts	0.99 [0.86–1.12]	0.83	1.19 [1.04–1.36]	9.45 ​× ​10^−3^	1.15 [1.01–1.31]	0.04	1.08 [0.94–1.23]	0.27
Heavy manual work	1.16 [1.08–1.25]	3.96 ​× ​10^−5^	1.23 [1.14–1.32]	3.40 ​× ​10^−8^	1.24 [1.15–1.33]	7.12 ​× ​10^−9^	1.10 [1.01–1.20]	0.03
Non-sedentary work	1.26 [1.19–1.34]	5.51 ​× ​10^−15^	1.24 [1.17–1.31]	1.34 ​× ​10^−12^	1.26 [1.18–1.33]	5.04 ​× ​10^−14^	1.21 [1.13–1.29]	1.18 ​× ​10^−7^
**Females**								
Shift work	1.04 [0.95–1.13]	0.43	1.11 [1.01–1.21]	0.03	1.06 [0.96–1.16]	0.25	1.00 [0.91–1.10]	0.95
Night shifts	0.99 [0.85–1.16]	0.90	1.08 [0.93–1.27]	0.31	1.01 [0.86–1.19]	0.88	0.95 [0.81–1.11]	0.53
Heavy manual work	1.14 [1.05–1.24]	1.53 ​× ​10^−3^	1.20 [1.11–1.30]	1.38 ​× ​10^−5^	1.19 [1.09–1.29]	5.53 ​× ​10^−5^	1.14 [1.04–1.25]	4.75 ​× ​10^−3^
Non-sedentary work	1.14 [1.08–1.20]	2.53 ​× ​10^−6^	1.10 [1.04–1.16]	8.64 ​× ​10^−4^	1.10 [1.04–1.16]	7.82 ​× ​10^−4^	1.06 [1.00–1.13]	0.06

Adjusted accordingly: Model 1 – unadjusted, Model 2 – age and sex, Model 3 – age, sex, BMI and TDI, Model 4 – age, sex, BMI, TDI and other employment factors.

a Denotes a sex interaction term with *P*-value <0.1.

Mutual adjustment (model 4) further attenuated these associations towards the null (shift work: OR 1.01 [95 ​% CI: 0.95–1.08], night shifts: 1.03 [0.93–1.14]). In sex-stratified analyses, a similar lack of association was seen between hip osteoarthritis and shift work in both males and females (Table 3, Supplementary Fig. 3). In combined sex analyses, associations between work physicality and hip osteoarthritis remained. Heavy manual work showed comparable associations with hip osteoarthritis in females and males, while sex interactions remained for prolonged non-sedentary work, with stronger associations in males (Table 3, Supplementary Fig. 3). When educational attainment was included as a covariate in sensitivity analysis, similar effect estimates were observed. (Supplementary Table 2).

**Fig. 2:**
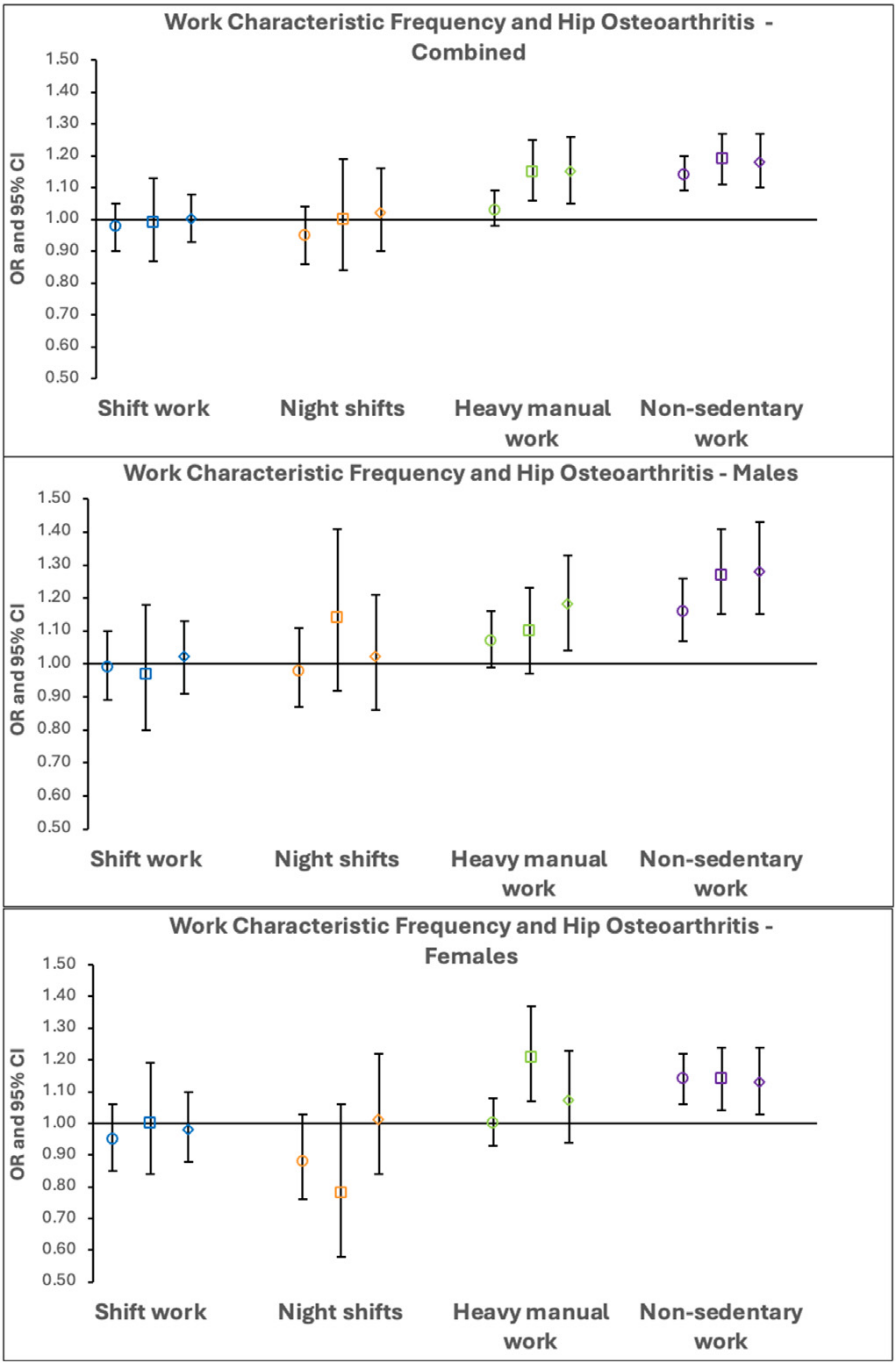
**Logistic regression results for the associations between categorical work characteristic frequency and hip osteoarthritis in fully-adjusted combined and sex-stratified analyses.** Odds ratios with the comparator group “never” are displayed with 95 ​% CIs. Different shapes represent the various employment intensities - circle: “sometimes”, square: “usually” and diamond: “always”.

In categorical analyses, as with knee osteoarthritis, there was no indication of a progressive positive association between both more frequent shift work and nights shifts and hip osteoarthritis. Similar results were seen in sex stratified analysis (Fig. 2, Supplementary Table 4). A more progressive association was notable between both heavy manual work and non-sedentary work frequency and hip osteoarthritis. Stronger associations were evident in males. Interestingly, the progressive positive association between work physicality frequency and risk of hip osteoarthritis was absent in females (Fig. 2, Supplementary Table 4).

### 3.4 Self-reported osteoarthritis

In combined sex analyses (models 1–3) associations between binary shift work and self-reported osteoarthritis were present across all models (model 3: OR 1.23 [95 ​% CI: 1.17–1.30]). Similar patterns emerged in night shift analyses. For both, sex interactions were present in model 3 and sex-stratified analyses showed stronger associations in males (Table 4, Supplementary Fig. 4). In combined sex analyses, unadjusted for shift work, heavy manual work and prolonged non-sedentary work showed strong associations with self-reported osteoarthritis. Sex interactions were observed in all work physicality models and in sex stratified analyses males exhibited higher odds of self-reported osteoarthritis (Table 4, Supplementary Fig. 4).

**Table 4:** Logistic regression results showing the associations between binary work exposures and self-reported osteoarthritis in combined and sex-stratified analyses.

Self-reported osteoarthritis	Model 1		Model 2		Model 3		Model 4	
	OR [95 ​% CI]	P	OR [95 ​% CI]	P	OR [95 ​% CI]	P	OR [95 ​% CI]	P
**Combined**								
Shift work	1.15 [1.09–1.21]	6.83 ​× ​10^−8^	1.32 [1.25–1.39]	1.16 ​× ​10^−26^	1.23 [1.17–1.30]	1.51 ​× ​10^−15^^a^	1.13 [1.07–1.19]	5.54 ​× ​10^−6^^a^
Night shifts	1.11 [1.03–1.20]	6.20 ​× ​10^−3^	1.37 [1.27–1.48]	3.56 ​× ​10^−15^	1.26 [1.16–1.36]	1.22 ​× ​10^−8^^a^	1.15 [1.06–1.24]	7.00 ​× ​10^−4^^a^
Heavy manual work	1.21 [1.16–1.26]	1.21 ​× ​10^−17^^a^	1.36 [1.30–1.42]	7.30 ​× ​10^−43^^a^	1.34 [1.28–1.40]	2.95 ​× ​10^−38^^a^	1.20 [1.14–1.27]	5.54 ​× ​10^−13^^a^
Non-sedentary work	1.26 [1.22–1.30]	5.30 ​× ​10^−46^^a^	1.24 [1.20–1.28]	4.96 ​× ​10^−38^^a^	1.24 [1.20–1.28]	2.87 ​× ​10^−37^^a^	1.15 [1.10–1.19]	1.27 ​× ​10^−12^^a^
**Males**								
Shift work	1.19 [1.11–1.28]	3.64 ​× ​10^−6^	1.38 [1.28–1.49]	2.78 ​× ​10^−17^	1.31 [1.22–1.42]	2.82 ​× ​10^−12^	1.20 [1.11–1.30]	3.77 ​× ​10^−6^
Night shifts	1.16 [1.04–1.29]	5.85 ​× ​10^−3^	1.39 [1.25–1.55]	2.65 ​× ​10^−9^	1.31 [1.17–1.46]	1.35 ​× ​10^−6^	1.19 [1.07–1.33]	1.78 ​× ​10^−3^
Heavy manual work	1.35 [1.27–1.44]	8.48 ​× ​10^−23^	1.43 [1.34–1.52]	3.35 ​× ​10^−30^	1.42 [1.34–1.51]	4.80 ​× ​10^−29^	1.21 [1.12–1.30]	2.94 ​× ​10^−7^
Non-sedentary work	1.41 [1.34–1.49]	1.00 ​× ​10^−40^	1.39 [1.32–1.46]	1.57 ​× ​10^−36^	1.39 [1.32–1.46]	4.00 ​× ​10^−36^	1.26 [1.19–1.34]	7.61 ​× ​10^−14^
**Females**								
Shift work	1.17 [1.09–1.25]	4.84 ​× ​10^−6^	1.25 [1.17–1.34]	1.15 ​× ​10^−10^	1.16 [1.08–1.24]	3.00 ​× ​10^−5^	1.08 [1.00–1.16]	0.04
Night shifts	1.20 [1.07–1.34]	1.35 ​× ​10^−3^	1.32 [1.18–1.48]	1.64 ​× ​10^−6^	1.18 [1.06–1.33]	4.03 ​× ​10^−3^	1.09 [0.97–1.23]	0.15
Heavy manual work	1.22 [1.15–1.30]	3.33 ​× ​10^−10^	1.29 [1.21–1.37]	4.64 ​× ​10^−15^	1.26 [1.18–1.34]	3.19 ​× ​10^−12^	1.17 [1.09–1.26]	1.42 ​× ​10^−5^
Non-sedentary work	1.18 [1.14–1.23]	2.16 ​× ​10^−15^	1.15 [1.10–1.19]	3.68 ​× ​10^−10^	1.14 [1.10–1.19]	7.26 ​× ​10^−10^	1.08 [1.03–1.14]	1.07 ​× ​10^−3^

Adjusted accordingly: Model 1 – unadjusted, Model 2 – age and sex, Model 3 – age, sex, BMI and TDI, Model 4 – age, sex, BMI, TDI and other employment factors.

a Denotes a sex interaction term with *P*-value <0.1.

Consistent with other osteoarthritis outcomes, mutual adjustment for the other work factors (model 4) weakened the associations between binary work type and self-reported osteoarthritis. In combined sex analyses, shift work and night shifts retained associations with self-reported osteoarthritis (shift work: OR 1.13 [95 ​% CI: 1.07–1.19], night shifts: 1.15 [1.06–1.24]). Sex interactions persisted for both and they remained more strongly associated with self-reported osteoarthritis in males than females (Table 4). Work physicality maintained its associations with self-reported osteoarthritis (heavy manual work: 1.20 [1.14–1.27], prolonged non-sedentary work: 1.15 [1.10–1.19]). Sex interactions remained and again stronger associations were seen between work physicality and self-reported osteoarthritis in males than females (Table 4, Supplementary Fig. 4). Furthermore, adding educational attainment to model 4 did little to attenuate the relationships seen (Supplementary Table 2).

**Fig. 3:**
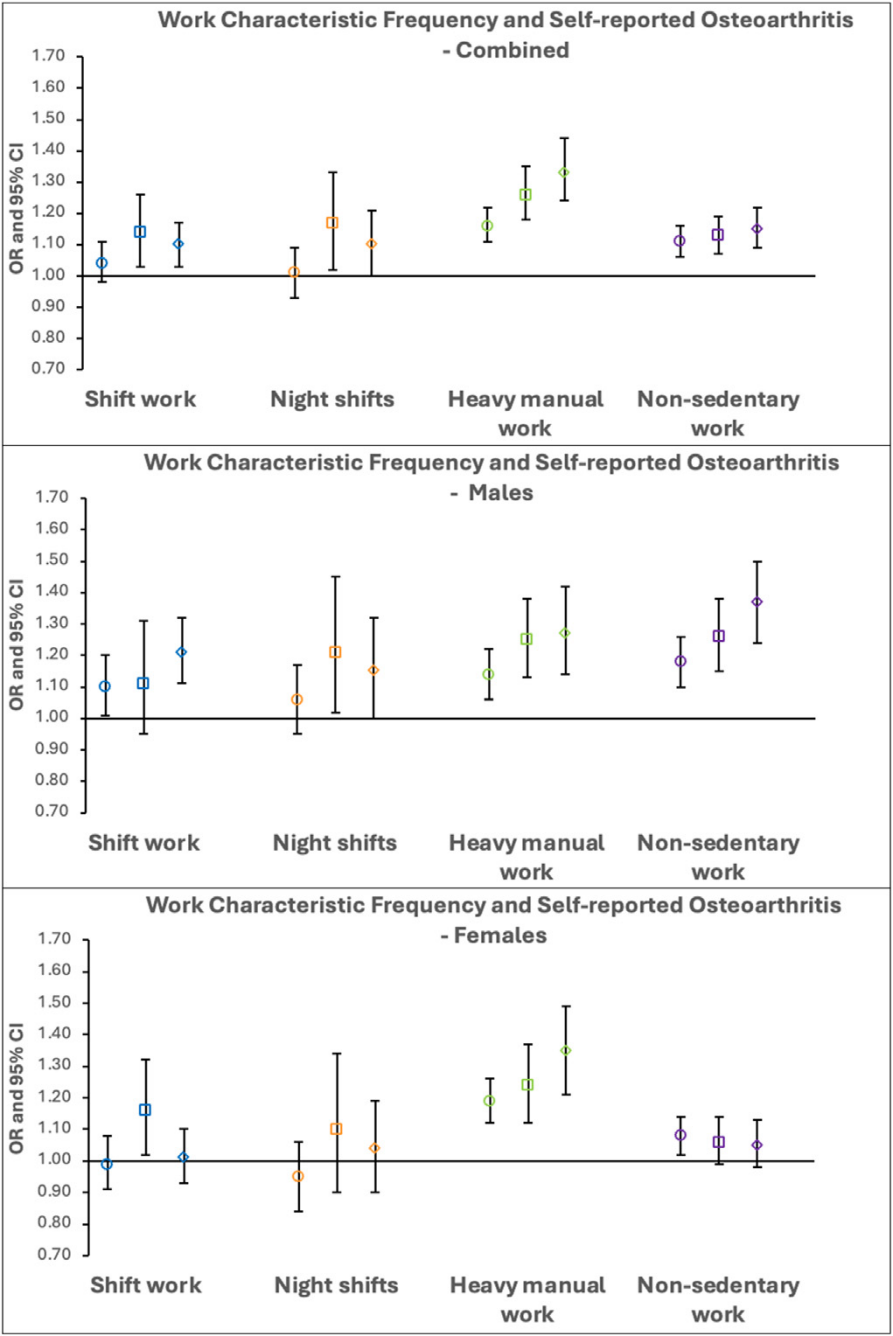
**Logistic regression results for the associations between categorical work characteristic frequency and self-reported osteoarthritis in fully-adjusted combined and sex-stratified analyses.** Odds ratios with the comparator group “never” are displayed with 95 ​% CIs. Different shapes represent the various employment intensities - circle: “sometimes”, square: “usually” and diamond: “always”.

Overall, in fully adjusted categorical analyses of shift work and night shift frequency, there were no progressive associations with self-reported osteoarthritis. However, progressive associations were observed for work physicality (Fig. 3, Supplementary Table 5). Unlike knee and hip osteoarthritis, increasing heavy manual work frequency saw progressive associations with self-reported osteoarthritis in females. In contrast, for prolonged non-sedentary work, progressive associations were seen in males but not females (Fig. 3, Supplementary Table 5).

## 4 Discussion

As far as we are aware, this is the largest cross-sectional study to date exploring the relationships between work characteristics (i.e. shift work, night-shifts, heavy manual work and prolonged non-sedentary work) and osteoarthritis (knee, hip and self-reported). Overall, both shift work and night shifts were associated with increased risk of knee osteoarthritis and self-reported osteoarthritis, while no such associations were observed for hip osteoarthritis. Importantly, these associations remained after adjustment for age, sex, BMI and deprivation, as well as the physical nature of work. Greater associations were seen between work physicality and osteoarthritis compared to shift work and night shifts, which were stronger in the knee than the hip. Broadly, work characteristics showed larger association with osteoarthritis in males as compared with females. The increasing frequency of heavy manual work and prolonged non-sedentary work showed clear progressive trends with an increasing risk of osteoarthritis. Shift work and night shifts did not display a similar trend.

Previous epidemiological research has linked shift work to an increased risk of osteoarthritis, in smaller Chinese cohorts. One study saw a hazard ratio of 1.19 for knee osteoarthritis in shift workers compared to non-shift workers, after adjusting for work posture but also working years [13]. Another study saw a comparable OR of 1.22 for lower-limb osteoarthritis in shift workers compared to day workers, after adjusting for work posture [14]. In comparison, this study saw an OR of 1.12 for both shift work and night shifts with knee osteoarthritis, after adjusting for work physicality. The lack of a progressive association between increasing frequency of shift work or night shifts with risk of osteoarthritis could be attributed to the limited number of responses in the “*usually*” group, which underpowered these analyses. Nevertheless, progressive positive associations were present in physicality analyses, suggesting these associations are weaker for shift work. Another explanation is circadian adaptation among more permanent shift workers. However, research indicates that less than 3 ​% show complete adjustment with only 25 ​% experiencing a beneficial degree of adjustment. Therefore, it is unlikely that most workers undergo sufficient synchronisation to benefit their health [26].

Although causal associations cannot be inferred, these findings are supported by animal studies, where circadian rhythm disruption, whether through environmental disturbance or genetic deletion of clock genes, contributed to osteoarthritis-like changes [11,27]. While underlying mechanisms remain unclear, shift work is an established cause of circadian misalignment, due to frequent alterations in sleep-wake and light-dark cycles and has been shown to desynchronise clock gene expression [28]. Therefore, shift work may be viewed as a proxy for circadian rhythm disruption and these findings provide evidence that circadian rhythm dysfunction is an important risk factor for osteoarthritis. Interestingly, genetic knockout animal studies only saw degenerative cartilage changes in the knee joint and not at the hip, as was seen in this study [27]. Since work physicality was adjusted for, this suggests that the knee may be more vulnerable to the effects of circadian rhythm disruption, independent of more extreme physical stressors. Indeed existing research has indicated disparities in the cellular and molecular pathophysiology of knee and hip osteoarthritis, including differences in inflammatory processes [29], but further research is required to elucidate any differences within the context of shift work and circadian rhythm disruption.

Both heavy manual work and prolonged non-sedentary work exposures demonstrated associations with all osteoarthritis outcomes. Occupational physical stressors and risk of osteoarthritis have been more extensively explored. For instance, a longitudinal cohort study found heavy manual work was associated with an increased risk of knee osteoarthritis, double that of sedentary work [30]. In another cross-sectional study, workers conducting highly physical work exhibited a nearly two-fold higher prevalence of symptomatic osteoarthritis, compared to those with less physical work [31]. Multiple reviews have also concluded that occupations characterised by heavy physical workloads, repetitive actions and prolonged walking and standing are associated with increased risk of knee and hip osteoarthritis [[32], [33], [34]].

Although heavy manual work and prolonged non-sedentary work showed associations with both knee and hip osteoarthritis, these were stronger at the knee. A different study also saw a greater association between job-related physical activity and knee osteoarthritis, with a hazard ratio of 1.39 compared with 1.22 for hip osteoarthritis [35]. This may be explained by the increased susceptibility of the knee to biomechanical stress. During everyday movements the knee experiences higher peak compressive forces compared to the hip [36]. Moreover, as the knee has more restricted movement and less stability, it is more inclined to injury, which can accelerate degenerative changes [29,37].

Despite associations being present for both males and females, work physicality mostly displayed stronger associations with osteoarthritis in males. Likewise, a meta-analysis of 71 studies found greater odds of knee osteoarthritis in workers with physically demanding occupations, that were male (OR 1.61), compared to female (1.35) [32]. Across all occupations, men demonstrate a stronger correlation between physical stressors, repetitive tasks, work demands and risk of injury [38]. Moreover, in hip osteoarthritis analyses, the progressive associations between work frequency and risk of osteoarthritis were not observed in females and similar findings have been reported in other studies where positive associations were solely found in males [39]. These differences could be attributed to the greater engagement in manual labour among the male demographic, reflected by the larger number of male-only studies [39], or the possibility that men may be more likely to engage in physically demanding work for longer periods, but more work is justified to fully understand the reasons behind this.

This study has identified robust associations between work-related exposures and osteoarthritis, which have important implications for occupational health policies. Further research is needed to establish causal relationships through randomised trials of workplace interventions. For example, therapies and interventions that tackle sleep hygiene, shift timing and light exposures have demonstrated favourable results in improving sleep among shift workers and may have a positive impact on joint health [40]. Considering that certain occupational exposures are likely unavoidable, it may be more feasible to examine dose-response associations to identify thresholds of safe working and to introduce closer monitoring or screening of musculoskeletal health in these workers. Another area of interest is the chronotherapeutic administration of prospective disease-modifying therapies and it may be useful to explore differences in medication timing between day and night shift workers [41].

A notable strength of this study is its large sample size, enhancing the statistical power of the analyses. Mutual-adjustment allowed for the estimation of independent associations between shift work and work physicality with risk of osteoarthritis, to be drawn, addressing a gap in prior research. UKB contains extensive lifestyle data, enabling adjustments for age, sex, BMI and deprivation, which have been associated with osteoarthritis and employment. Previously, socioeconomic status has been largely unconsidered [13,14], but was included in this study.

A main limitation of this study is its cross-sectional nature, preventing the inference of causal associations in isolation. While UKB collects data on job type and duration of workplace exposures, the information was not sufficiently detailed to support analyses of the influence of frequency, intensity or duration of work-place patterns on subsequent osteoarthritis diagnosis. Regarding shift work, the triangulation between this study and animal studies does nonetheless add to the causal hypothesis that circadian rhythm dysfunction causes knee osteoarthritis. Another limitation is that UKB predominantly comprises Caucasian participants and those who tend to be more healthy and less deprived, restricting generalisability to other ethnicities. Further research among more ethnically diverse cohorts would be beneficial. Furthermore, rather than a mediator, BMI may act as a collider since both employment type and osteoarthritis could increase BMI, introducing collider bias if adjusted for. However, the decrease in effect estimates on adjustment suggests no spurious associations were induced. Another limitation is the large number of individuals (43.1 ​%) excluded from this analysis due to missing data, which might cause a selection bias. Although, our sample size is still unprecedented. Finally, it was not possible to distinguish between participants with osteoarthritis at one joint and those who developed incidental osteoarthritis at another joint, but this bias would most likely reduce effect sizes rather than introduce spurious results.

In conclusion, this large cross-sectional study saw independent associations between shift work and both knee and self-reported osteoarthritis, but not hip osteoarthritis. These findings support the role of circadian rhythm dysfunction as a contributing factor in knee osteoarthritis pathogenesis, which aligns with the results of previous animal studies. Although further replication of these findings is warranted given the duration of work exposures were not known. Work physicality demonstrated association with osteoarthritis at all sites, with the strongest associations seen in the knee, possibly reflecting its higher susceptibility to biomechanical stress. These findings highlight the importance of the workplace environment in risk factor management and could inform strategies to promote joint health in the workforce. Further research is warranted to explore the effects of workplace interventions in minimising the risk of osteoarthritis and to investigate the anatomical discrepancies that were observed.

## Author contributions

Significant contributions were made by all authors towards the conception and design of this study, the acquisition of data, its analysis and interpretation. Each author helped draft the article before agreeing the final version of this manuscript. Asad Hashmi (tc20694@bristol.ac.uk) takes full responsibility for the integrity of the work.

## Role of the funding source

AH and SS were self-funded undergraduate students. BGF is supported by an 10.13039/501100000272NIHR Academic Clinical Lectureship and an 10.13039/501100000691Academy of Medical Sciences Starter Grant (SGL030∖1057). RB and MJ were supported by a 10.13039/100010269Wellcome Trust collaborative award (209233/Z/17/Z). QJM was supported by a 10.13039/501100012041Versus Arthritis Senior Fellowship Award 20875.

## Conflict of interests

None to declare.
