# Supplementary material for: Associations between work characteristics and osteoarthritis: A cross-sectional study of 285,947 UK Biobank participants
Source: Osteoarthr Cartil Open. 2025 Jan 10;7(1):100565. doi: 10.1016/j.ocarto.2025.100565 (PMC11803847; doi:10.1016/j.ocarto.2025.100565)
Supplement: Multimedia component 1 [file mmc1.docx]

**Supplementary**

**Supplementary Table 1 - ICD 9 and 10 codes for osteoarthritis**

|  | **ICD 10 codes** | | **ICD 9 codes** | |
| --- | --- | --- | --- | --- |
| **Knee Osteoarthritis** | **M17** | Gonarthrosis [arthrosis of knee] | **71536** | Unspecific localised osteoarthrosis/allied disorder (lower leg) |
|  | **M170** | Primary gonarthrosis, bilateral |  |  |
|  | **M171** | Other primary gonarthrosis |  |  |
|  | **M179** | Gonarthrosis, unspecified | **71516** | Localised, primary osteoarthrosis and allied disorders (lower leg) |
|  | **M1906** | Primary arthrosis of other joints (lower leg) |  |  |
|  | **M1996** | Arthrosis, unspecified (lower leg) |  |  |
| **Hip Osteoarthritis** | **M16** | Coxarthrosis [arthrosis of hip] | **71535** | Unspecified localised osteoarthrosis/allied disorder (pelvic region and thigh) |
|  | **M160** | Primary coxarthrosis, bilateral |  |  |
|  | **M161** | Other primary coxarthrosis |  |  |
|  | **M169** | Coxarthrosis, unspecified | **71515** | Localised primary osteoarthrosis/allied disorder (pelvic region and thigh) |
|  | **M1905** | Primary arthrosis of other joints (pelvic region and thigh) |  |  |
|  | **M1995** | Arthrosis, unspecified (pelvic region and thigh) |  |  |

*Table adapted from Zengini et al. (20)*

| **Knee Osteoarthritis** | **Shift Work** | | | | **Night Shifts** | | | | **Heavy Manual Work** | | | | **Non-sedentary Work** | | | |
| --- | --- | --- | --- | --- | --- | --- | --- | --- | --- | --- | --- | --- | --- | --- | --- | --- |
|  | **Unadjusted** | | **Fully Adjusted** | | **Unadjusted** | | **Fully Adjusted** | | **Unadjusted** | | **Fully Adjusted** | | **Unadjusted** | | **Fully Adjusted** | |
|  | OR [95%CI] | P | OR [95%CI] | P | OR [95%CI] | P | OR [95%CI] | P | OR [95%CI] | P | OR [95%CI] | P | OR [95%CI] | P | OR [95%CI] | P |
| **Combined** | | | | | | | | | | | | | | | | |
| **Sometimes** | 1.25 [1.19-1.32] | 4.08 × 10^-17^ | 1.08 [1.03-1.15] | 3.89 × 10^-3^ | 1.32 [1.24-1.40] | 9.52 × 10^-19^ | 1.16 [1.09-1.23] | 8.28 × 10^-6^ | 1.41 [1.36-1.46] | 1.50 × 10^-78^ | 1.17 [1.12-1.22] | 3.77 × 10^-13^ | 1.31 [1.26-1.36] | 1.10 × 10^-40^ | 1.17 [1.13-1.22] | 4.46 × 10^-14^ |
| **Usually** | 1.31 [1.20-1.44] | 9.49 × 10^-9^ | 1.08 [0.99-1.19] | 9.76 × 10^-2^ | 1.17 [1.04-1.32] | 9.67 × 10^-3^ | 1.01 [0.89-1.15] | 0.85 | 1.65 [1.56-1.74] | 6.20 × 10^-77^ | 1.34 [1.26-1.42] | 6.97 × 10^-20^ | 1.58 [1.51-1.66] | 4.40 × 10^-85^ | 1.33 [1.26-1.40] | 3.21 × 10^-26^ |
| **Always** | 1.34 [1.28-1.41] | 1.26 × 10^-30^ | 1.11 [1.05-1.17] | 2.87 × 10^-4^ | 1.44 [1.32-1.56] | 3.86 × 10^-18^ | 1.16 [1.06-1.26] | 7.55 × 10^-4^ | 1.77 [1.68-1.86] | 8.00 × 10^-103^ | 1.45 [1.36-1.55] | 1.19 × 10^-28^ | 1.79 [1.72-1.87] | 2.00 × 10^-169^ | 1.38 [1.31-1.46] | 3.88 × 10^-31^ |
| **Males** | | | | | | | | | | | | | | | | |
| **Sometimes** | 1.23 [1.15-1.32] | 2.87 × 10^-9^ | 1.07 [1.00-1.15] | 0.05 | 1.29 [1.19-1.39] | 1.63 × 10^-10^ | 1.17 [1.08-1.27] | 1.02 × 10^-4^ | 1.51 [1.44-1.59] | 6.20 × 10^-59^ | 1.21 [1.14-1.28] | 3.35 × 10^-10^ | 1.43 [1.35-1.51] | 7.41 × 10^-34^ | 1.25 [1.18-1.33] | 4.78 × 10^-13^ |
| **Usually** | 1.15 [1.01-1.31] | 0.04 | 1.01 [0.89-1.16] | 0.84 | 1.17 [1.01-1.35] | 0.04 | 1.09 [0.94-1.27] | 0.27 | 1.85 [1.73-1.97] | 5.10 × 10^-73^ | 1.45 [1.34-1.58] | 6.30 × 10^-20^ | 1.90 [1.78-2.02] | 1.00 × 10^-84^ | 1.50 [1.39-1.61] | 4.48 × 10^-26^ |
| **Always** | 1.29 [1.21-1.38] | 5.12 × 10^-14^ | 1.14 [1.06-1.23] | 2.24 × 10^-4^ | 1.32 [1.19-1.46] | 3.08 × 10^-7^ | 1.17 [1.05-1.30] | 5.60 × 10^-3^ | 1.92 [1.80-2.05] | 1.20 × 10^-84^ | 1.58 [1.45-1.72] | 2.11 × 10^-25^ | 2.10 [1.98-2.23] | 2.00 × 10^-133^ | 1.55 [1.43-1.67] | 3.36 × 10^-27^ |
| **Females** | | | | | | | | | | | | | | | | |
| **Sometimes** | 1.24 [1.14-1.34] | 2.23 × 10^-7^ | 1.11 [1.02-1.20] | 0.02 | 1.30 [1.17-1.44] | 8.08 × 10^-7^ | 1.15 [1.03-1.28] | 9.81 × 10^-3^ | 1.29 [1.23-1.36] | 7.47 × 10^-23^ | 1.13 [1.06-1.20] | 6.67 × 10^-5^ | 1.21 [1.14-1.27] | 3.18 × 10^-11^ | 1.12 [1.06-1.19] | 1.53 × 10^-4^ |
| **Usually** | 1.49 [1.31-1.70] | 1.57 × 10^-9^ | 1.20 [1.05-1.38] | 8.26 × 10^-3^ | 1.09 [0.88-1.35] | 0.43 | 0.90 [0.72-1.12] | 0.34 | 1.32 [1.21-1.45] | 1.23 × 10^-9^ | 1.13 [1.01-1.25] | 0.03 | 1.29 [1.21-1.38] | 3.60 × 10^-14^ | 1.18 [1.09-1.27] | 1.70 × 10^-5^ |
| **Always** | 1.37 [1.27-1.48] | 7.84 × 10^-16^ | 1.09 [1.01-1.19] | 0.04 | 1.57 [1.38-1.79] | 1.46 × 10^-11^ | 1.17 [1.02-1.34] | 0.03 | 1.50 [1.37-1.63] | 9.41 × 10^-20^ | 1.22 [1.10-1.36] | 2.54 × 10^-4^ | 1.53 [1.44-1.62] | 1.30 × 10^-46^ | 1.27 [1.17-1.36] | 8.67 × 10^-10^ |

*Odds ratios are in comparison to the “Never” group.*

*The fully adjusted model is adjusted for age, sex, BMI, TDI and other employment factors.*

**Supplementary Table 1 - Logistic regression results showing the associations between categorical work frequency and knee osteoarthritis in combined and sex-stratified analyses.**

**S*upplementary Table 3 - Logistic regression results showing the associations between categorical work frequency and hip osteoarthritis in combined and sex-stratified analyses.***

*Odds ratios are in comparison to the “Never” group.*

*The fully adjusted model is adjusted for age, sex, BMI, TDI and other employment factors.*

| **Hip Osteoarthritis** | **Shift Work** | | | | **Night Shifts** | | | | **Heavy Manual Work** | | | | **Non-sedentary Work** | | | |
| --- | --- | --- | --- | --- | --- | --- | --- | --- | --- | --- | --- | --- | --- | --- | --- | --- |
|  | **Unadjusted** | | **Fully Adjusted** | | **Unadjusted** | | **Fully Adjusted** | | **Unadjusted** | | **Fully Adjusted** | | **Unadjusted** | | **Fully Adjusted** | |
|  | OR [95%CI] | P | OR [95%CI] | P | OR [95%CI] | P | OR [95%CI] | P | OR [95%CI] | P | OR [95%CI] | P | OR [95%CI] | P | OR [95%CI] | P |
| **Combined** | | | | | | | | | | | | | | | | |
| **Sometimes** | 0.96 [0.90-1.04] | 0.34 | 0.98 [0.90-1.05] | 0.54 | 0.87 [0.79-0.96] | 3.66 × 10^-3^ | 0.95 [0.86-1.04] | 0.26 | 1.11 [1.06-1.17] | 8.96 × 10^-6^ | 1.03 [0.98-1.09] | 0.24 | 1.21 [1.15-1.27] | 3.59 × 10^-14^ | 1.14 [1.09-1.20] | 2.66 × 10^-7^ |
| **Usually** | 1.05 [0.92-1.19] | 0.49 | 0.99 [0.87-1.13] | 0.85 | 0.91 [0.76-1.08] | 0.26 | 1.00 [0.84-1.19] | 0.97 | 1.19 [1.11-1.29] | 1.75 × 10^-6^ | 1.15 [1.06-1.25] | 1.24 × 10^-3^ | 1.30 [1.22-1.38] | 8.90 × 10^-18^ | 1.19 [1.11-1.27] | 2.37 × 10^-7^ |
| **Always** | 0.97 [0.90-1.04] | 0.40 | 1.00 [0.93-1.08] | 0.96 | 0.99 [0.88-1.12] | 0.91 | 1.02 [0.90-1.16] | 0.70 | 1.13 [1.04-1.21] | 1.97 × 10^-3^ | 1.15 [1.05-1.26] | 3.60 × 10^-3^ | 1.32 [1.25-1.39] | 1.30 × 10^-23^ | 1.18 [1.10-1.27] | 2.27 × 10^-6^ |
| **Males** | | | | | | | | | | | | | | | | |
| **Sometimes** | 1.00 [0.90-1.11] | 0.98 | 0.99 [0.89-1.10] | 0.88 | 0.93 [0.82-1.04] | 0.21 | 0.98 [0.87-1.11] | 0.75 | 1.22 [1.14-1.31] | 1.49 × 10^-8^ | 1.07 [0.99-1.16] | 0.09 | 1.27 [1.18-1.37] | 2.77 × 10^-10^ | 1.16 [1.07-1.26] | 1.83 × 10^-4^ |
| **Usually** | 0.97 [0.80-1.18] | 0.77 | 0.97 [0.80-1.18] | 0.77 | 1.03 [0.83-1.27] | 0.77 | 1.14 [0.92-1.41] | 0.24 | 1.23 [1.12-1.36] | 3.63 × 10^-5^ | 1.10 [0.97-1.23] | 0.13 | 1.43 [1.31-1.57] | 1.13 × 10^-15^ | 1.27 [1.15-1.41] | 3.68 × 10^-6^ |
| **Always** | 0.96 [0.87-1.06] | 0.41 | 1.02 [0.91-1.13] | 0.77 | 0.95 [0.81-1.12] | 0.57 | 1.02 [0.86-1.21] | 0.81 | 1.24 [1.12-1.37] | 2.56 × 10^-5^ | 1.18 [1.04-1.33] | 0.01 | 1.44 [1.32-1.56] | 5.87 × 10^-18^ | 1.28 [1.15-1.43] | 6.63 × 10^-6^ |
| **Females** | | | | | | | | | | | | | | | | |
| **Sometimes** | 0.95 [0.85-1.06] | 0.35 | 0.95 [0.85-1.06] | 0.36 | 0.83 [0.72-0.97] | 0.02 | 0.88 [0.76-1.03] | 0.11 | 1.05 [0.98-1.12] | 0.17 | 1.00 [0.93-1.08] | 0.97 | 1.18 [1.11-1.26] | 6.44 × 10^-7^ | 1.14 [1.06-1.22] | 2.20 × 10^-4^ |
| **Usually** | 1.13 [0.95-1.34] | 0.17 | 1.00 [0.84-1.19] | 0.98 | 0.77 [0.57-1.03] | 0.08 | 0.78 [0.58-1.06] | 0.11 | 1.24 [1.11-1.38] | 1.26 × 10^-4^ | 1.21 [1.07-1.37] | 2.55 × 10^-3^ | 1.21 [1.12-1.31] | 3.45 × 10^-6^ | 1.14 [1.04-1.24] | 4.28 × 10^-3^ |
| **Always** | 1.00 [0.91-1.11] | 0.94 | 0.98 [0.88-1.10] | 0.75 | 1.09 [0.91-1.31] | 0.33 | 1.01 [0.84-1.22] | 0.88 | 1.07 [0.95-1.20] | 0.28 | 1.07 [0.94-1.23] | 0.31 | 1.25 [1.16-1.34] | 2.51 × 10^-9^ | 1.13 [1.03-1.24] | 9.88 × 10^-3^ |

| **Self-reported Osteoarthritis** | **Shift Work** | | | | **Night Shifts** | | | | **Heavy Manual Work** | | | | **Non-sedentary Work** | | | |
| --- | --- | --- | --- | --- | --- | --- | --- | --- | --- | --- | --- | --- | --- | --- | --- | --- |
|  | **Unadjusted** | | **Fully Adjusted** | | **Unadjusted** | | **Fully Adjusted** | | **Unadjusted** | | **Fully Adjusted** | | **Unadjusted** | | **Fully Adjusted** | |
|  | OR [95%CI] | P | OR [95%CI] | P | OR [95%CI] | P | OR [95%CI] | P | OR [95%CI] | P | OR [95%CI] | P | OR [95%CI] | P | OR [95%CI] | P |
| **Combined** | | | | | | | | | | | | | | | | |
| **Sometimes** | 1.06 [1.00-1.12] | 0.06 | 1.04 [0.98-1.11] | 0.16 | 0.95 [0.89-1.03] | 0.20 | 1.01 [0.93-1.09] | 0.86 | 1.24 [1.19-1.29] | 8.89 × 10^-29^ | 1.16 [1.11-1.22] | 1.32 × 10^-11^ | 1.20 [1.15-1.25] | 2.98 × 10^-18^ | 1.11 [1.06-1.16] | 1.72 × 10^-6^ |
| **Usually** | 1.25 [1.14-1.39] | 6.58 × 10^-6^ | 1.14 [1.03-1.26] | 0.01 | 1.09 [0.96-1.24] | 0.20 | 1.17 [1.02-1.33] | 0.02 | 1.27 [1.20-1.35] | 4.44 × 10^-15^ | 1.26 [1.18-1.35] | 4.43 × 10^-11^ | 1.31 [1.24-1.37] | 4.99 × 10^-27^ | 1.13 [1.07-1.19] | 1.97 × 10^-5^ |
| **Always** | 1.12 [1.06-1.19] | 4.44 × 10^-5^ | 1.10 [1.03-1.17] | 2.39 × 10^-3^ | 1.12 [1.02-1.24] | 0.02 | 1.10 [1.00-1.21] | 0.06 | 1.29 [1.22-1.37] | 4.01 × 10^-17^ | 1.33 [1.24-1.44] | 1.22 × 10^-14^ | 1.44 [1.38-1.50] | 8.10 × 10^-60^ | 1.15 [1.09-1.22] | 1.45 × 10^-6^ |
| **Males** | | | | | | | | | | | | | | | | |
| **Sometimes** | 1.16 [1.07-1.26] | 6.21 × 10^-4^ | 1.10 [1.01-1.20] | 0.03 | 1.06 [0.96-1.17] | 0.24 | 1.06 [0.95-1.17] | 0.28 | 1.36 [1.28-1.44] | 1.55 × 10^-23^ | 1.14 [1.06-1.22] | 3.05 × 10^-4^ | 1.33 [1.24-1.42] | 1.94 × 10^-16^ | 1.18 [1.10-1.26] | 4.77 × 10^-6^ |
| **Usually** | 1.17 [1.00-1.37] | 0.05 | 1.11 [0.95-1.31] | 0.19 | 1.18 [0.99-1.40] | 0.07 | 1.21 [1.02-1.45] | 0.03 | 1.50 [1.38-1.63] | 7.39 × 10^-22^ | 1.25 [1.13-1.38] | 1.03 × 10^-5^ | 1.54 [1.43-1.67] | 1.13 × 10^-27^ | 1.26 [1.15-1.38] | 4.86 × 10^-7^ |
| **Always** | 1.22 [1.12-1.33] | 2.04 × 10^-6^ | 1.21 [1.11-1.32] | 1.66 × 10^-5^ | 1.16 [1.02-1.33] | 0.03 | 1.15 [1.00-1.32] | 0.05 | 1.48 [1.36-1.61] | 5.16 × 10^-20^ | 1.27 [1.14-1.42] | 8.87 × 10^-6^ | 1.72 [1.60-1.85] | 1.40 × 10^-50^ | 1.37 [1.24-1.50] | 7.44 × 10^-11^ |
| **Females** | | | | | | | | | | | | | | | | |
| **Sometimes** | 1.04 [0.96-1.13] | 0.32 | 0.99 [0.91-1.08] | 0.88 | 0.96 [0.86-1.07] | 0.46 | 0.95 [0.84-1.06] | 0.34 | 1.23 [1.17-1.29] | 3.80 × 10^-16^ | 1.19 [1.12-1.26] | 3.41 × 10^-9^ | 1.17 [1.11-1.23] | 2.00 × 10^-9^ | 1.08 [1.02-1.14] | 4.71 × 10^-3^ |
| **Usually** | 1.36 [1.20-1.55] | 1.88 × 10^-6^ | 1.16 [1.02-1.32] | 0.03 | 1.15 [0.94-1.39] | 0.17 | 1.10 [0.90-1.34] | 0.37 | 1.25 [1.14-1.36] | 6.70 × 10^-7^ | 1.24 [1.12-1.37] | 2.64 × 10^-5^ | 1.22 [1.14-1.30] | 8.38 × 10^-10^ | 1.06 [0.99-1.14] | 0.09 |
| **Always** | 1.12 [1.03-1.21] | 4.82 × 10^-3^ | 1.01 [0.93-1.10] | 0.85 | 1.23 [1.07-1.41] | 3.29 × 10^-3^ | 1.04 [0.90-1.19] | 0.63 | 1.32 [1.21-1.44] | 1.85 × 10^-10^ | 1.35 [1.21-1.49] | 1.89 × 10^-8^ | 1.32 [1.25-1.39] | 4.33 × 10^-22^ | 1.05 [0.98-1.13] | 0.19 |

*Odds ratios are in comparison to the “Never” group.*

*The fully adjusted model is adjusted for age, sex, BMI, TDI and other employment factors.*

**S*upplementary Table 4 - Logistic regression results showing the associations between categorical work frequency and self-reported osteoarthritis in combined and sex-stratified analyses.***

| Knee OA | Model 4 | | Model 4 + Education | |
| --- | --- | --- | --- | --- |
|  | **OR [95% CI]** | **P** | **OR [95% CI]** | **P** |
| Shift work | 1.12 [1.07-1.17] | 3.87 × 10^-6^ | 1.09 [1.04–1.16] | 1.00 x 10^-3^ |
| Night shifts | 1.12 [1.04-1.20] | 1.75 × 10^-3^ | 1.12 [1.03–1.21] | 5.00 x 10^-3^ |
| Heavy manual work | 1.30 [1.24-1.36] | 5.76 × 10^-30^ | 1.25 [1.19-1.32] | 5.00 x 10^-17^ |
| Non-sedentary work | 1.32 [1.28-1.37] | 5.30 × 10^-54^ | 1.29 [1.24-1.34] | 1.60 x 10^-36^ |
| Hip OA | Model 4 | | Model 4 + Education | |
|  | **OR [95% CI]** | **P** | **OR [95% CI]** | **P** |
| Shift work | 1.01 [0.95-1.08] | 0.74 | 0.99 [0.92–1.08] | 0.95 |
| Night shifts | 1.03 [0.93-1.14] | 0.59 | 1.05[0.93–1.17] | 0.45 |
| Heavy manual work | 1.13 [1.06-1.20] | 1.32 × 10^-4^ | 1.14 [1.06-1.23] | 4.90 x 10^-4^ |
| Non-sedentary work | 1.12 [1.07-1.17] | 1.39 × 10^-6^ | 1.12 [1.06-1.18] | 1.50 x 10^-5^ |
| Self-reported OA | Model 4 | | Model 4 + Education | |
|  | **OR [95% CI]** | **P** | **OR [95% CI]** | **P** |
| Shift work | 1.13 [1.07-1.19] | 5.54 × 10^-6^ | 1.10 [1.05–1.18] | 5.90 x 10^-4^ |
| Night shifts | 1.15 [1.06-1.24] | 7.00 × 10^-4^ | 1.12[1.02–1.22] | 0.02 |
| Heavy manual work | 1.20 [1.14-1.27] | 5.54 × 10^-13^ | 1.20 [1.13-1.28] | 1.30 x 10^-9^ |
| Non-sedentary work | 1.15 [1.10-1.19] | 1.27 × 10^-12^ | 1.14 [1.09-1.18] | 2.40 x 10^-9^ |

**S*upplementary Table 5 - Logistic regression results showing the effects of the addition of education as a covariate to the associations between binary work ezposures and osteoarthritisoutcomes in combined sex analyses.***

*Existing results from model 4 (adjusted for age, sex, BMI, TDI and other employment factors) are presented compared with results from a further regression model including educational attainment (defined through a categorical variable recording highest education certificate: 1- College or University degree, 2 – AS/A Levels or equivalent, 3 - GCSEs/O Levels or equivalent, 4 – CSEs or equivalent, 5 - NVQs/HND/HNC or equivalent, 6 – other professional qualifications e.g. nursing or teaching).*

**UK Biobank Cohort**

N = 502,366

**Missing HES osteoarthritis data**: 3

**Osteoarthritis Data Available**

N = 502,363

**Prefer not to answer/do not know:** 1,170

**Missing employment data**: 213,662

**Employment Data Available**

N = 287,531

**Missing BMI data**: 1,091*

**Missing TDI data**: 495*

**Confounder Data Available**

N = 285,947

**Supplementary Figure 1 –** Flow chart to visualise the exclusion and inclusion of participants from the UK Biobank study.

**2 participants were missing both BMI and TDI*


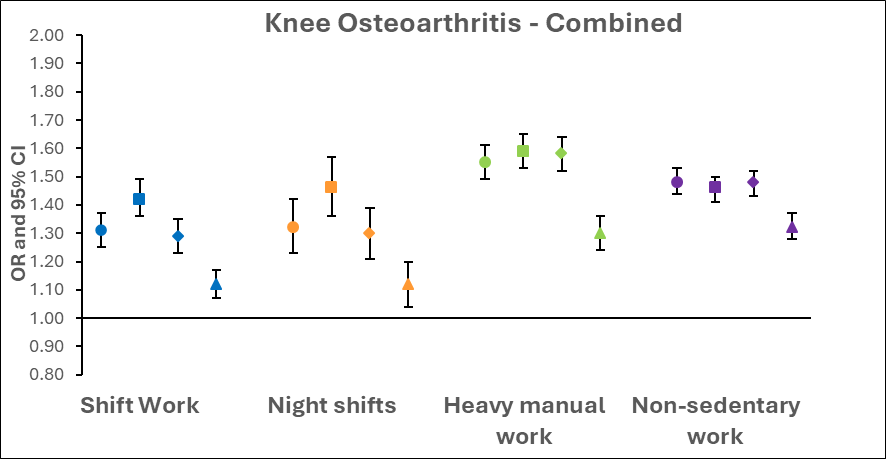

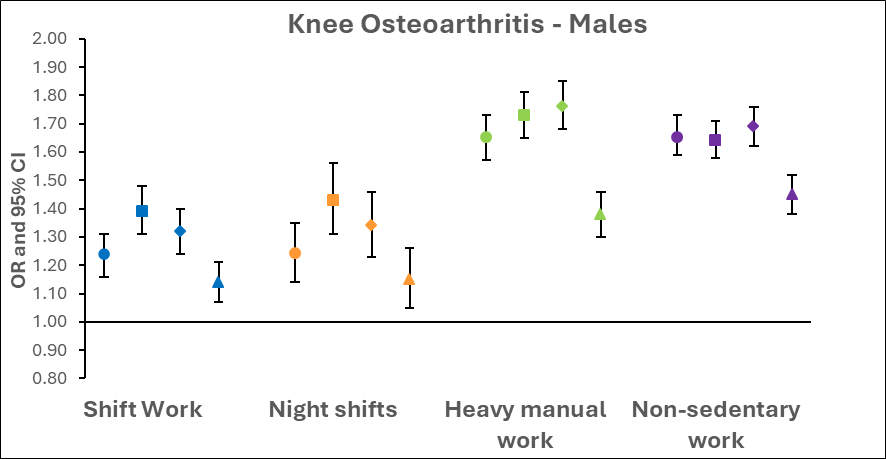

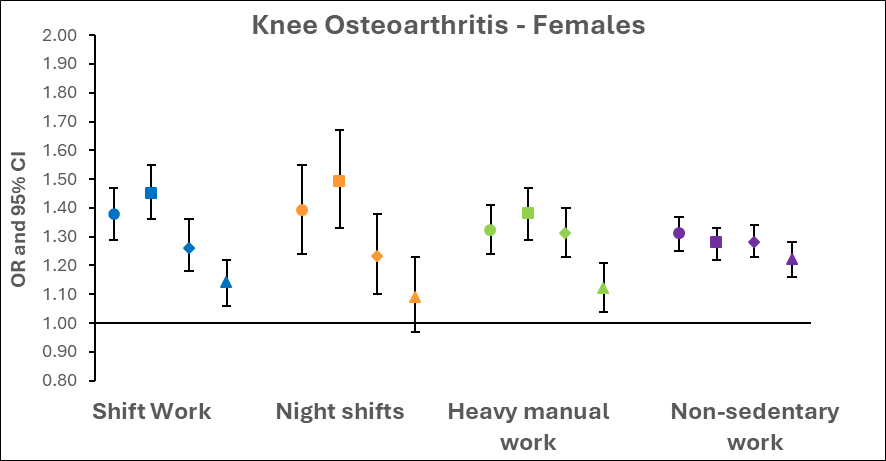


**Supplementary Figure 2 – Logistic regression results for the associations between binary work exposures and knee osteoarthritis in combined and sex-stratified analyses.**

*Odds ratios with 95% confidence intervals displayed. Different shapes represent the various adjustments - circle: unadjusted (model 1), square: age and sex (model 2), diamond: age, sex, BMI and TDI (model 3) and triangle: age, sex, BMI, TDI and other work variables (model 4).*


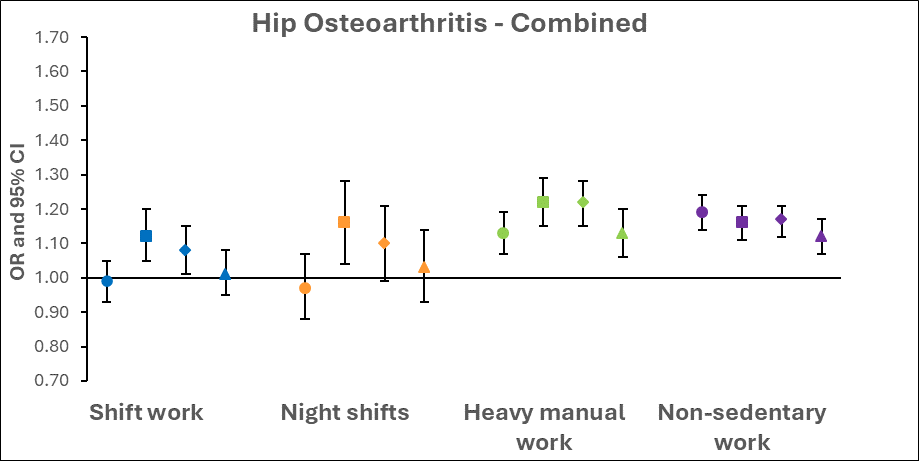

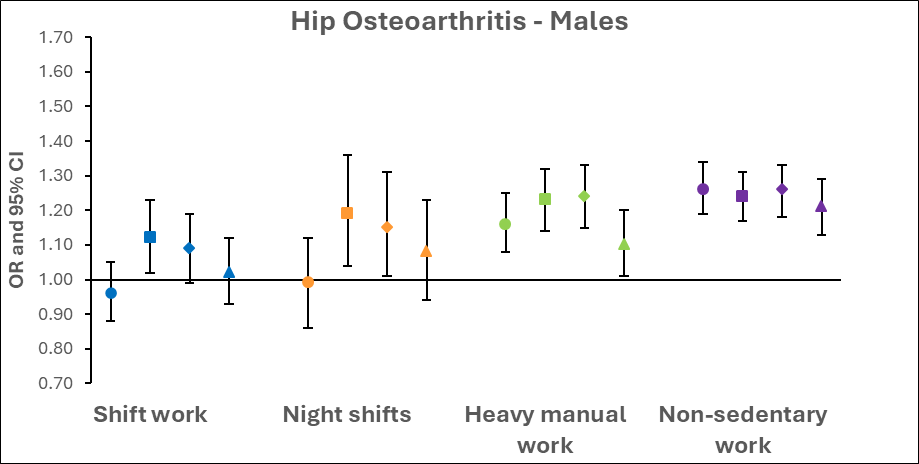

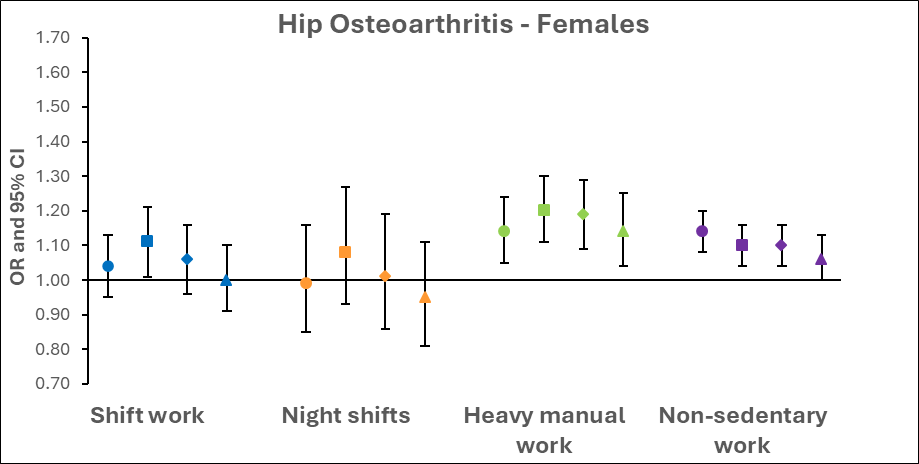


**Supplementary Figure 3 – Logistic regression results for the associations between binary work exposures and hip osteoarthritis in combined and sex-stratified analyses.**

*Odds ratios with 95% confidence intervals displayed. Different shapes represent the various adjustments - circle: unadjusted (model 1), square: age and sex (model 2), diamond: age, sex, BMI and TDI (model 3) and triangle: age, sex, BMI, TDI and other work variables (model 4).*


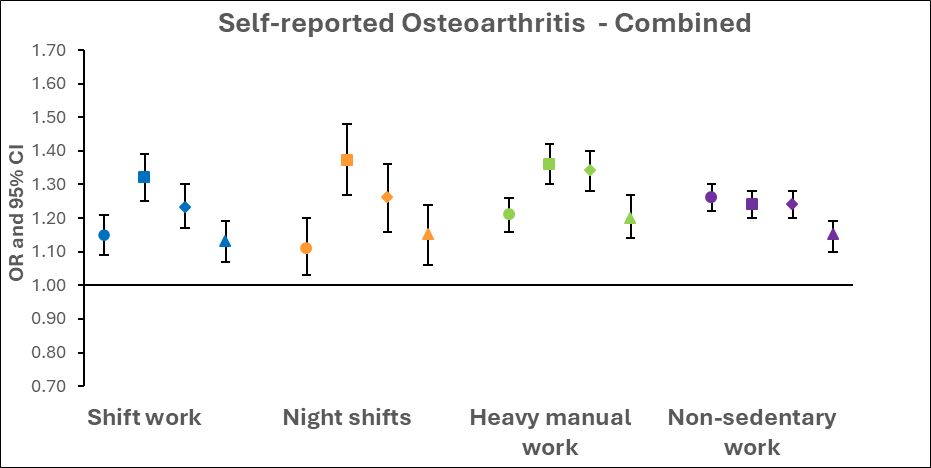

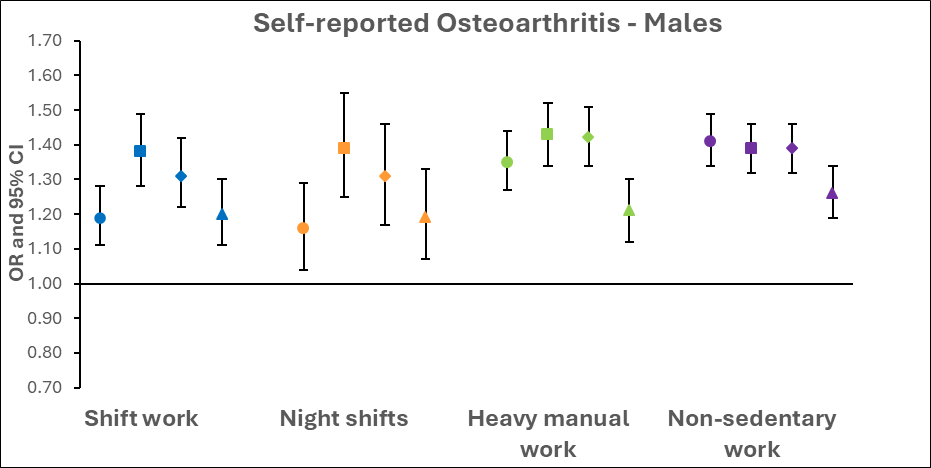

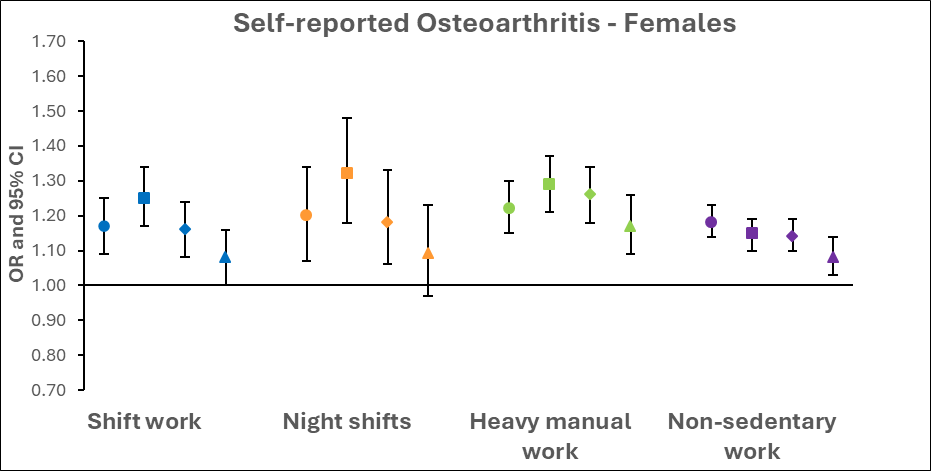


**Supplementary Figure 4 – Logistic regression results for the associations between binary work exposures and self-reported osteoarthritis in combined and sex-stratified analyses.**

*Odds ratios with 95% confidence intervals displayed. Different shapes represent the various adjustments - circle: unadjusted (model 1), square: age and sex (model 2), diamond: age, sex, BMI and TDI (model 3) and triangle: age, sex, BMI, TDI and other work variables (model 4).*
