# Supplementary material for: Associations between work characteristics and osteoarthritis: A cross-sectional study of 285,947 UK Biobank participants
Source: Osteoarthr Cartil Open. 2025 Jan 10;7(1):100565. doi: 10.1016/j.ocarto.2025.100565 (PMC11803847; doi:10.1016/j.ocarto.2025.100565)
Supplement: Multimedia component 2 [file mmc2.docx]

**Title**

Describing the associations between employment patterns and osteoarthritis: a cross-sectional study from UK Biobank

**Background**

Osteoarthritis (OA) is a prevalent disorder of the synovial joints encompassing mechanical, inflammatory and metabolic factors (1). Abnormalities in the anabolic and catabolic balance of cartilage tissue have been recognised in the pathogenesis of OA (2). Chronic overloading and joint overuse have been attributed to this disruption in chondrocyte homeostasis, with physical workload identified as a common occupational risk factor for OA, notably in the weight-bearing joints (3). Research has indicated that heavy manual occupations are associated with a two-fold higher risk of knee OA, particularly in men, perhaps due to greater engagement in manual labour among the male population (4). However, many studies exploring this association are outdated and restricted by their sample size. More recently, connections between circadian rhythm disruption, at a cellular level, and OA have been explored. The “cartilage clock” refers to the diurnal variation in cartilage tissue regulated by clock genes and endogenous pacemakers (5). Shift work induced circadian misalignment has emerged as a risk factor for chronic metabolic diseases including OA (6), but there is limited research in the UK exploring this association or research focused on joints besides the knee. With an increasing demand for shift work and a growing prevalence of OA, it is vital further research is undertaken in larger cohorts to investigate associations between employment patterns and OA.

This project will focus on observing work shift patterns and work physicality within UK Biobank, a large prospective cohort of ~500,000 participants initiated in 2006. Participants were asked at baseline and at three follow-up appointments, about the physicality of their current and previous employment and whether this involved shift work. Further information on lifestyle and phenotypic measures, such as BMI, were obtained and participants provided consent to data linkage with hospital episode statistics. Once identified, the associations between these employment patterns and OA of the knee and hip will be explored using self-reported and hospital diagnosed ICD outcomes. Furthermore, previously derived radiographic OA data for a subsample of the cohort, will enable comparisons to be drawn based on radiographically-defined OA outcomes (7). A more comprehensive understanding of the relationship between employment risk factors and development of OA may help prevent disease onset and progression and contribute to the development of therapies which acknowledge the role of the circadian rhythm.

**Research Question**

What are the associations between employment patterns, including shift work and physicality of work, and osteoarthritis of the hip and knee?

**Aims and objectives:**

1. Describe employment patterns in UK Biobank
2. Identify any associations between engaging in shift work and risk of developing OA.
3. Identifying the extent to which physical work contributes to risk of OA.
4. Explore any differences in relationships between shift work, physical work and risk of OA based on anatomical site.

**Exposures**

- Job involves night shift work any time (usually or always across B/L – F3).
- Job involves shift work any time (usually or always across B/L – F3)
- Job involved heavy manual or physical work (usually or always across B/L – F3).
- Job involves mainly walking or standing (usually or always across B/L – F3).

**Outcomes**

- Patient-reported OA (non-site specific)
- Hospital diagnosed OA of the hip and/or knee (derived from ICD-9/10 codes)
- Radiographic OA of the hip and/or knee (grades 1-4)

**Covariates (Adjusted for)**

- Age (years)
- Sex
- BMI (kg/m^2^)
- Socioeconomic status (TDI)
- Manual work (for shift work) and night shift work (for work physicality)
- (Smoking?)

**Inclusion**

- Primary population: all those who had answered the question: "Does your work involve shift work?" and the work physicality questions.
- Subsamples: all those with rOA of the hip and/or knee (derived from DXA).

**Analysis Strategy**

- Descriptive statistics (mean, SD, range) to analyse population characteristics (age, height, weight, BMI, sex, SES).
- Descriptive statistics (mean, SD, range) to explore the distribution of shift work and work physicality variables.
- Standardise exposures.
- Logistic Regression
  - Exposures: Job involves night shift work, shift work, heavy manual/physical work and mainly walking/standing.
  - Outcomes: patient-reported OA, presence of radiographic OA (grade ≥2), hsopital diagnosed OA.
- Multi-nomial logistic Regression
  - Exposures: Job involves night shift work, shift work, heavy manual/physical work and mainly walking/standing.
  - Outcomes: radiographic OA grades 1-4

**Sensitivity Analyses**

Are grades ≥3 radiographic OA associated more with employment variables than grades <3?

**DAG for Shift work and OA**

Age

Sex

SES

BMI

**Exposure – Circadian Rhythm dysfunction**

Proxied by: (Night) Shift Work

**Outcome – Osteoarthritis**

Proxied by: Self-reported OA, Hospital diagnosed OA of hip +/or knee, rOA of hip +/or knee,

Work Physicality

**DAG for Work physicality and OA**

Age

Sex

SES

BMI

**Outcome - Osteoarthritis**

Proxied by: Self-reported OA, Hospital diagnosed OA of hip +/or knee, rOA of hip +/or knee,

**Exposures**

Heavy manual/physical work

Mainly walking/standing work

Shift work

**Reference List**

1. He Y, Li Z, Alexander PG, Ocasio-Nieves BD, Yocum L, Lin H, et al. Pathogenesis of Osteoarthritis: Risk Factors, Regulatory Pathways in Chondrocytes, and Experimental Models. Biology (Basel). 2020;9(8).

2. Fujii Y, Liu L, Yagasaki L, Inotsume M, Chiba T, Asahara H. Cartilage Homeostasis and Osteoarthritis. Int J Mol Sci. 2022;23(11).

3. Yucesoy B, Charles LE, Baker B, Burchfiel CM. Occupational and genetic risk factors for osteoarthritis: a review. Work. 2015;50(2):261-73.

4. Perry TA, Wang X, Gates L, Parsons CM, Sanchez-Santos MT, Garriga C, et al. Occupation and risk of knee osteoarthritis and knee replacement: A longitudinal, multiple-cohort study. Seminars in Arthritis and Rheumatism. 2020;50(5):1006-14.

5. Rogers N, Meng Q-J. Tick tock, the cartilage clock. Osteoarthritis and Cartilage. 2023;31(11):1425-36.

6. Zhou M, Yang S, Guo Y, Wang D, Qiu W, Wang B, et al. Shift work and the risk of knee osteoarthritis among Chinese workers: a retrospective cohort study. Scandinavian Journal of Work, Environment & Health. 2020(2):152-60.

7. Faber BG, Ebsim R, Saunders FR, Frysz M, Lindner C, Gregory JS, et al. A novel semi-automated classifier of hip osteoarthritis on DXA images shows expected relationships with clinical outcomes in UK Biobank. Rheumatology (Oxford). 2022;61(9):3586-95.
